# External Urethral Sphincter Pressure Measurement: An Accurate Method for the Diagnosis of Detrusor External Sphincter Dyssynergia?

**DOI:** 10.1371/journal.pone.0037996

**Published:** 2012-05-31

**Authors:** Carlos H. Suzuki Bellucci, Jens Wöllner, Flavia Gregorini, Dorothee Birnböck, Marko Kozomara, Ulrich Mehnert, Thomas M. Kessler

**Affiliations:** Neuro-Urology, Spinal Cord Injury Center & Research, Balgrist University Hospital, University of Zürich, Zürich, Switzerland; University of Adelaide, Australia

## Abstract

**Background:**

Combined pelvic floor electromyography (EMG) and videocystourethrography (VCUG) during urodynamic investigation are the most acceptable and widely agreed methods for diagnosing detrusor external sphincter dyssynergia (DESD). Theoretically, external urethral sphincter pressure (EUSP) measurement would provide enough information for the diagnosis of DESD and could simplify the urodynamic investigation replacing combined pelvic floor EMG and VCUG. Thus, we evaluated the diagnostic accuracy of EUSP measurement for DESD.

**Patients & Methods:**

A consecutive series of 72 patients (36 women, 36 men) with neurogenic lower urinary tract dysfunction able to void spontaneously was prospectively evaluated at a single university spinal cord injury center. Diagnosis of DESD using EUSP measurement (index test) versus combined pelvic floor EMG and VCUG (reference standard) was assessed according to the recommendations of the Standards for Reporting of Diagnostic Accuracy Initiative.

**Results:**

Using EUSP measurement (index test) and combined pelvic floor EMG and VCUR (reference standard), DESD was diagnosed in 10 (14%) and in 41 (57%) patients, respectively. More than half of the patients presented discordant diagnosis between the index test and the reference standard. Among 41 patients with DESD diagnosed by combined pelvic floor EMG and VCUR, EUSP measurement identified only 6 patients. EUSP measurement had a sensitivity of 15% (95% CI 5%–25%), specificity of 87% (95% CI 76%–98%), positive predictive value of 60% (95% CI 30%–90%), and negative predictive value of 56% (95% CI 44%–68%) for the diagnosis of DESD.

**Conclusions:**

For diagnosis of DESD, EUSP measurement is inaccurate and cannot replace combined pelvic floor EMG and VCUR.

## Introduction

Detrusor external sphincter dyssynergia (DESD) is defined as a detrusor contraction concurrent with an involuntary contraction of the urethral and/or periurethral striated muscle [1]. It usually occurs due to neurological lesions below the pontine micturition center and above the sacral cord, i.e. lesions interrupting spinobulbar pathways [2]. As both the bladder and the external urethral sphincter contracts simultaneously, high voiding pressure and large post void residual may lead to life-threatening complications such as recurrent urinary tract infections with septicemia, vesico-uretero-renal reflux, hydronephrosis, and renal failure [3], [4].

Despite the high clinical relevance of DESD, there is no single “gold standard” method for its diagnosis. Blaivas and Fisher [5] proposed the combination of pelvic floor electromyography (EMG) and videocystourethrography (VCUG) during urodynamic investigation in order to achieve the highest accuracy level for the diagnosis of DESD. Indeed, these combined examinations are still the most acceptable and widely agreed diagnostic method. However, urethral pressure measurement at the site of the external sphincter during urodynamic investigation would theoretically provide enough information for the diagnosis of DESD [6] and the use of a multiple transducer catheter measuring intravesical and urethral pressure simultaneously [7] would simplify the urodynamic investigation.

We hypothesized that the measurement of external urethral sphincter pressure (EUSP) could replace combined pelvic floor EMG and VCUR for the diagnosis of DESD. Thus, we prospectively evaluated the diagnostic accuracy of EUSP measurement for DESD.

## Patients and Methods

### Ethics statement

This study was approved by the local ethics committee of the University of Zürich (i.e. the Kantonale Ethikkommission Zürich, Switzerland, study identification number: EK 2010-0207/0) and registered with ClinicalTrials.gov (study registration number: NCT01293110). All participants gave written informed consent.

### Patients

From November 2010 to April 2011, 191 consecutive patients older than 18 years with neurogenic lower urinary tract dysfunction (NLUTD) underwent video-urodynamic investigation at the Spinal Cord Injury Center, Balgrist University Hospital, Zürich, Switzerland. Of those, 76 (39%) could void spontaneously and were prospectively enrolled into the study.

All methods, definitions, and units are according to the standards recommended by the International Continence Society [1]. In addition, as a study of diagnostic accuracy this article complies with the recommendations of the Standards for Reporting of Diagnostic Accuracy Initiative [8].

### Measurements

**Figure 1:**
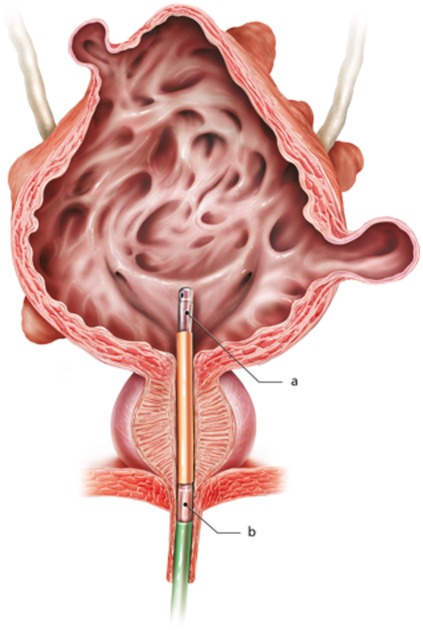
An 8 French transurethral reusable microtip dual sensor microtransducer catheter (Unisensor AG, Attikon, Switzerland) was used to simultaneously measure the intravesical and urethral pressure. The microtransducers were positioned in the bladder (a) and the external urethral sphincter (b) under the guidance of continuous pressure monitoring and fluoroscopy ensuring correct position during the urodynamic investigation.

Video-urodynamic investigations were performed according to Good Urodynamic Practices recommended by the International Continence Society [9]. All patients were urodynamically investigated in a sitting position. An 8 French transurethral reusable microtip dual sensor microtransducer catheter (Unisensor AG, Attikon, Switzerland) was used to simultaneously measure the intravesical and urethral pressure. The microtransducers were positioned in the bladder and the external urethral sphincter (Figure 1) under the guidance of continuous pressure monitoring and fluoroscopy ensuring correct position during the urodynamic investigation. The bladder was filled with a 36°C mixture of Ringer's lactate solution and contrast medium at a speed of 20 mL/min. Pelvic floor electromyography was performed with surface electrodes (Ambu®, NF-50-K/W/12, Malaysia). A Sedia-NT multichannel urodynamic system (Sedia®, Givisiez, Switzerland) was applied for all measurements.

Blood pressure and heart rate were measured at the beginning and the end of the video-urodynamic investigation which was interrupted immediately in the case of signs of autonomic dysreflexia.

According to the literature [5], DESD was defined as an increase in pelvic floor EMG activity during detrusor contraction in the absence of Valsalva's or Credé's maneuver and/or a dilated posterior urethra obstructed by the external urethral sphincter in VCUR. Concerning EUSP measurement, DESD was defined as any increase, maintenance, or decrease <10 cmH_2_O of EUSP during the voiding phase. During video-urodynamic investigations, EUSP measurement (index test) and combined pelvic floor EMG and VCUG (reference standard) were performed simultaneously.

All video-urodynamic investigations were assessed by two experienced urologists in consensus. Pelvic floor EMG and VCUG were interpreted blinded to the EUSP measurements and vice versa.

### Outcome measures

The outcome measure was the diagnosis of DESD using EUSP measurement (index test) versus combined pelvic floor EMG and VCUG (reference standard).

### Statistical analyses

Data were normally distributed and they are presented as mean ± standard deviation (SD). Sensitivity, specificity, and predictive values of EUSP measurements including 95% confidence intervals (CI) were calculated. Statistical analyses were performed using SPSS version 17.0 (SPSS Inc, Chicago, IL, USA).

## Results

Of the 76 eligible patients, 4 with unclear results due to technical problems were excluded leaving 72 patients for analysis. Thirty six (50%) were women and 36 (50%) men. Mean age was 52±14 years (range 19–80). The causes of NLUTD were spinal cord injury in 40 (55%), multiple sclerosis in 10 (14%), spinal stenosis in 4 (6%), cauda equina syndrome in 2 (3%), spina bifida in 2 (3%), disk hernia in 2 (3%), and others in 12 (16%).

All patients voided spontaneously and as an adjunct 9 (12%) relied on clean intermittent self-catheterization and 8 (11%) on an indwelling suprapubic catheter. Fifty one (71%) patients had no medication for lower urinary tract dysfunction, 15 (21%) were on antimuscarinics, and 7 (10%) on alpha-blockers.

Urodynamic findings are summarized in Table 1. Detrusor pressure during the storage and voiding phase and post void residual were relatively high, whereas maximum flow rate and voided volume were quite small. Detrusor overactivity was found in 39 (54%) patients and none presented vesico-uretero-renal reflux.

**Table 1:** Urodynamic parameters of the 72 patients included in the study.

	Mean ± SD (range)
**Filling cystometry**	
Maximum cystometric capacity (mL)	475±198 (95–960)
Maximum detrusor pressure during the storage phase (cmH_2_O)	51±37 (10–218)
Compliance (mL/cmH_2_O)	71±47 (10–220)
**Pressure-flow study**	
Maximum flow rate (mL/s)	9±6 (1–28)
Maximum detrusor pressure during the voiding phase (cmH_2_O)	84±27 (44–241)
Detrusor pressure during maximum flow rate (cmH_2_O)	57±34 (5–237)
Voided volume (mL)	265±189 (15–920)
Post void residual (mL)	221±200 (0–910)

SD: standard deviation.

**Figure 2:**
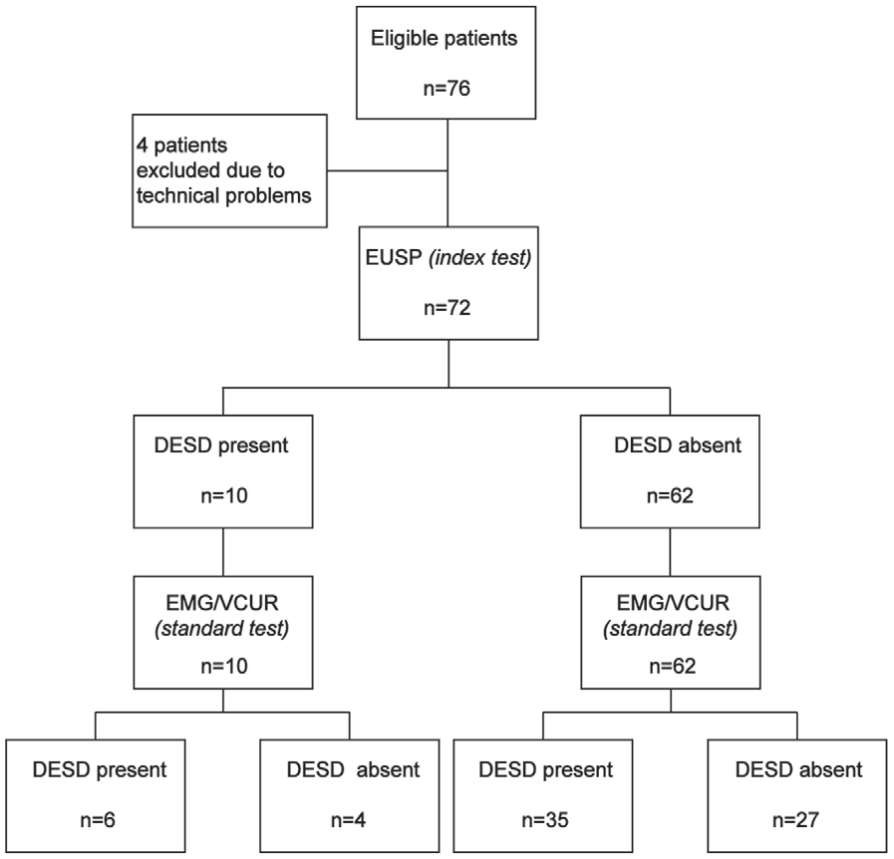
Standards for Reporting of Diagnostic Accuracy flow diagram. [8] Comparison of external urethral sphincter pressure (EUSP) measurement (index test) versus combined pelvic floor electromyography (EMG) and videocystourethrography (VCUR) (reference standard) for the diagnosis of detrusor external sphincter dyssynergia (DESD).

Using EUSP measurement (index test) and combined pelvic floor EMG and VCUR (reference standard), DESD was diagnosed in 10 (14%) and in 41 (57%) patients, respectively (Figure 2). More than half of the patients presented discordant diagnosis between the index test and the reference standard. Among 41 patients with DESD diagnosed by combined pelvic floor EMG and VCUR, EUSP measurement identified only 6 patients. EUSP measurement had a sensitivity of 15% (95% CI 5%–25%), specificity of 87% (95% CI 76%–98%), positive predictive value of 60% (95% CI 30%–90%), and negative predictive value of 56% (95% CI 44%–68%) for the diagnosis of DESD. In a gender sub-group analysis, we found similar results for female (Figure S1) and male (Figure S2) patients.

No adverse events related to the EUSP measurement (index test) and combined pelvic floor EMG and VCUR (reference standard) occurred during the study.

## Discussion

### Main findings

In the present study including 72 patients with NLUTD, EUSP measurements were inaccurate for the diagnosis of DESD. Thus, in contrast to our initial hypothesis, EUSP measurement cannot replace combined pelvic floor EMG and VCUR and is therefore not recommended to assess DESD.

### Findings in the context of existing evidence

Sakakibara et al. [10] evaluated video-urodynamically 44 patients with non-traumatic NLUTD. Among the patients with VCUR showing relaxation of bladder neck and external urethral sphincter besides disappearance of EMG activity during voiding phase, the mean reduction of EUSP was 39±25 cmH_2_O in women, 53±47 cmH_2_O in men, and >25 cmH_2_O in the majority of the patients. On the other side, among the patients who presented VCUR demonstrating incomplete/absent urethral opening at the external urethral sphincter site or increased/persistent EMG activity during the voiding phase, the mean reduction of EUSP was significantly smaller (6.4±6.7 cmH_2_O in women and 5.0±9.5 cmH_2_O in men). We therefore hypothesized that EUSP measurement could be an accurate method for the diagnosis of DESD.

Although the clinical and pathophysiological definition of DESD is standardized by the International Continence Society [9], the method for diagnosing DESD is not. There is little accuracy data for diagnosing DESD [11] and several authors reported various techniques for assessing DESD, especially in earlier work defining the field of neuro-urology [5], [6], [7], [12], [13]. Blaivas et al. [5] included VCUR as part of a complete urodynamic investigation and concluded that by measuring and displaying all the parameters simultaneously, a much clearer understanding of normal and abnormal physiology is obtained. Moreover, De et al. [11] reported that the concordance between VCUR and EMG for diagnosis of DESD is only 60% and affirmed that the combination of both methods may be advantageous in identifying DESD. We therefore considered combined pelvic floor EMG and VCUR as the reference standard to compare and estimate the accuracy of EUSP measurement. In contrast to De et al. [11], however, we used non-invasive surface electrodes instead of needle electrodes for patient comfort reasons.

### Implications for practice

With the advent of multiple transducer catheters, simultaneous measurement of urethral and intravesical pressure using the same catheter has become possible. This seems a promising method for the diagnosis of DESD since the urodynamic investigation may be relevantly shortened and simplified. In addition, replacing combined pelvic floor EMG and VCUR by EUSP for the diagnosis of DESD would reduce the investigative costs and also the radiation exposure during urodynamics. However, we found inacceptable accuracy of EUSP measurement for the diagnosis of DESD with a false negative rate of 85% and low positive and negative predictive values not supporting the use of this technique in daily clinical practice.

### Implications for research

It is generally agreed that urethral pressure is of significant value for lower urinary tract function [14]. However, although urethral pressure measurement is widely used, it still remains a challenge to define the optimal way to characterize, measure, and transform the findings usefully into daily clinical practice. Perhaps, it may be the consequence of the methodology adopted since microtip catheters do not measure the urethral pressure directly but rather the normal stress component on the surface of the transducer. This stress is due to the interaction between the urethral tissue and the transducer surface. Thus, we hypothesize that the urine flow between the urethral wall and the transducer could cause a decrease of the urethral pressure. This would be in line with our finding that 62 (86%) patients showed a decrease of ESUP of >10 cmH_2_O. Further research involving basic science and engineering technology is necessary in order to improve urethral pressure measurements.

### Limitations of the study

To the best of our knowledge, this is the first study investigating the accuracy of EUSP measurement for the diagnosis of DESD. Although our study complies with the recommendations of the Standards for Reporting of Diagnostic Accuracy Initiative [8], there are several limitations that should be addressed. Techniques of pelvic floor EMG vary considerably including surface electrodes, coaxial needle electrodes, concentric needle electrodes, wire electrodes, and others. Since we used surface electrodes to perform pelvic floor EMG, it is unclear whether our results could be extrapolated to EMG techniques applying other types of electrodes. Finally, other confounding factors comprise the urethral pressure measurement technique, especially including type, size, material, orientation, and position of the catheter as well as the urodynamic system used. All these parameters must be taken in account when transposing the data to clinical practice. Indeed, different catheter systems may yield completely different results [15]. However, the diagnosis of DESD does not rely on absolute pressure values but on the changes of sphincter activity, and different catheters should be equally sensitive to vast changes in pressure so that the catheter type should not be extremely important, provided the catheter is not too thick.

### Conclusions

For the diagnosis of DESD using EUSP measurement, we found sensitivity, specificity, positive and negative predictive values of 15%, 87%, 60%, and 56%, respectively. Thus, EUSP measurement is inaccurate and cannot replace combined pelvic floor EMG and VCUR to assess DESD.

## Supporting Information

Figure S1 **Female sub-group analysis: Using EUSP measurement (index test) and combined pelvic floor EMG and VCUR (reference standard), DESD was diagnosed in 5 (14%) and in 23 (64%) female patients, respectively.** More than 60% of the female patients presented discordant diagnosis between the index test and the reference standard. Among 23 female patients with DESD diagnosed by combined pelvic floor EMG and VCUR, EUSP measurement identified only 3 female patients. In females, EUSP measurement had a sensitivity of 13% (95% CI 4%–32%), specificity of 84% (95% CI 57%–95%), positive predictive value of 60% (95% CI 11%–96%), and negative predictive value of 35% (95% CI 19%–54%) for the diagnosis of DESD.
(TIF)

Figure S2 **Male sub-group analysis: Using EUSP measurement (index test) and combined pelvic floor EMG and VCUR (reference standard), DESD was diagnosed in 5 (14%) and in 18 (50%) male patients, respectively.** Almost half of the male patients presented discordant diagnosis between the index test and the reference standard. Among 18 male patients with DESD diagnosed by combined pelvic floor EMG and VCUR, EUSP measurement identified only 3 male patients. In males, EUSP measurement had a sensitivity of 16% (95% CI 5%–39%), specificity of 89% (95% CI 67%–97%), positive predictive value of 60% (95% CI 11%–96%), and negative predictive value of 51% (95% CI 33%–69%) for the diagnosis of DESD.
(TIF)
