# Supplementary figures and images for: External Urethral Sphincter Pressure Measurement: An Accurate Method for the Diagnosis of Detrusor External Sphincter Dyssynergia?
Source: PLoS One. 2012 May 31;7(5):e37996. doi: 10.1371/journal.pone.0037996 (PMC3365101; doi:10.1371/journal.pone.0037996)

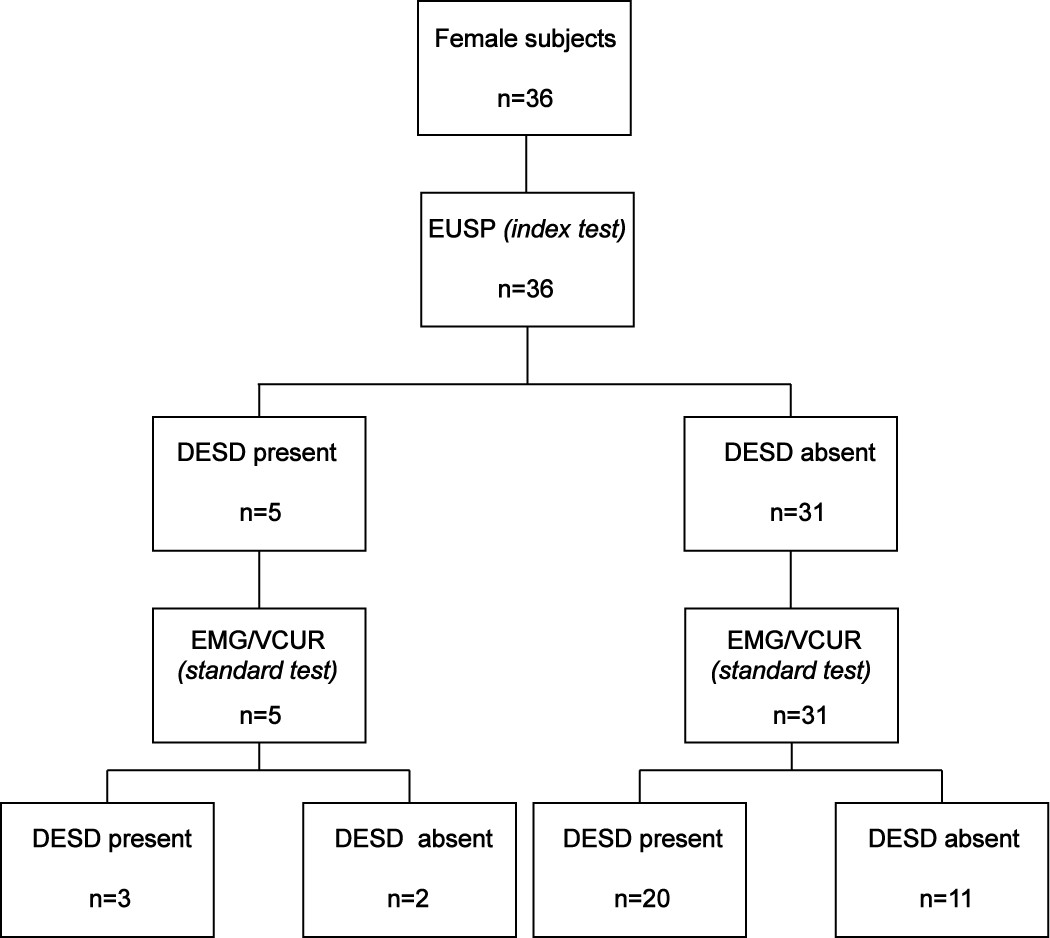

Supplement: Figure S1 — Female sub-group analysis: Using EUSP measurement (index test) and combined pelvic floor EMG and VCUR (reference standard), DESD was diagnosed in 5 (14%) and in 23 (64%) female patients, respectively. More than 60% of the female patients presented discordant diagnosis between the index test and the reference standard. Among 23 female patients with DESD diagnosed by combined pelvic floor EMG and VCUR, EUSP measurement identified only 3 female patients. In females, EUSP measurement had a sensitivity of 13% (95% CI 4%–32%), specificity of 84% (95% CI 57%–95%), positive predictive value of 60% (95% CI 11%–96%), and negative predictive value of 35% (95% CI 19%–54%) for the diagnosis of DESD. (TIF) [file pone.0037996.s001.tif]

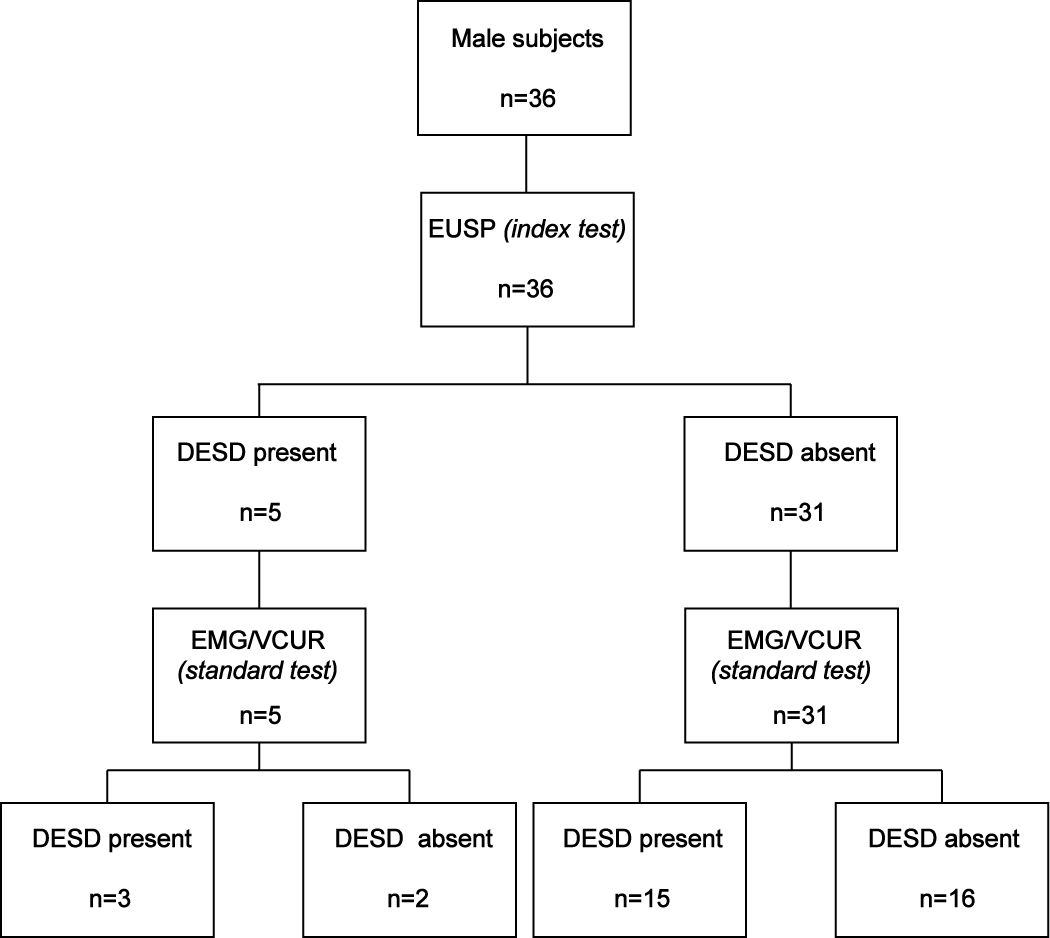

Supplement: Figure S2 — Male sub-group analysis: Using EUSP measurement (index test) and combined pelvic floor EMG and VCUR (reference standard), DESD was diagnosed in 5 (14%) and in 18 (50%) male patients, respectively. Almost half of the male patients presented discordant diagnosis between the index test and the reference standard. Among 18 male patients with DESD diagnosed by combined pelvic floor EMG and VCUR, EUSP measurement identified only 3 male patients. In males, EUSP measurement had a sensitivity of 16% (95% CI 5%–39%), specificity of 89% (95% CI 67%–97%), positive predictive value of 60% (95% CI 11%–96%), and negative predictive value of 51% (95% CI 33%–69%) for the diagnosis of DESD. (TIF) [file pone.0037996.s002.tif]
